# Determination of Anthracene on Ag-Au Alloy Nanoparticles/Overoxidized-Polypyrrole Composite Modified Glassy Carbon Electrodes

**DOI:** 10.3390/s101009449

**Published:** 2010-10-20

**Authors:** Stephen N. Mailu, Tesfaye T. Waryo, Peter M. Ndangili, Fanelwa R. Ngece, Abd A. Baleg, Priscilla G. Baker, Emmanuel I. Iwuoha

**Affiliations:** Sensor Lab, Department of Chemistry, University of the Western Cape, Bellville, 7535, South Africa; E-Mails: 2970836@uwc.ac.za (S.N.M.); twaryo@uwc.ac.za (T.W.); pndangili@uwc.ac.za (P.M.N.); fngece@uwc.ac.za (F.R.N.); baleg@uwc.ac.za (A.A.B.); eiwuoha@uwc.ac.za (E.I.I.)

**Keywords:** overoxidized-polypyrrole, bimetallic alloy nanoparticles, anthracene, electrocatalytic effect, composite, transmission electron microscopy

## Abstract

A novel electrochemical sensor for the detection of anthracene was prepared by modifying a glassy carbon electrode (GCE) with over-oxidized polypyrrole (PPyox) and Ag-Au (1:3) bimetallic nanoparticles (Ag-AuNPs). The composite electrode (PPyox/Ag-AuNPs/GCE) was prepared by potentiodynamic polymerization of pyrrole on GCE followed by its overoxidation in 0.1 M NaOH. Ag-Au bimetallic nanoparticles were chemically prepared by the reduction of AgNO_3_ and HAuCl_4_ using C_6_H_5_O_7_Na_3_ as the reducing agent as well as the capping agent and then immobilized on the surface of the PPyox/GCE. The nanoparticles were characterized by UV-visible spectroscopy technique which confirmed the homogeneous formation of the bimetallic alloy nanoparticles. Transmission electron microscopy showed that the synthesized bimetallic nanoparticles were in the range of 20–50 nm. The electrochemical behaviour of anthracene at the PPyox/Ag-AuNPs/GCE with Ag: Au atomic ratio 25:75 (1:3) exhibited a higher electrocatalytic effect compared to that observed when GCE was modified with each constituent of the composite (*i.e.*, PPyox, Ag-AuNPs) and bare GCE. A linear relationship between anodic current and anthracene concentration was attained over the range of 3.0 × 10^−6^ to 3.56 × 10^−4^ M with a detection limit of 1.69 × 10^−7^ M. The proposed method was simple, less time consuming and showed a high sensitivity.

## 1. Introduction

Polyaromatic hydrocarbons (PAHs) are chemical compounds that consist of fused aromatic rings and do not contain heteroatoms or carry substituents [1]. They occur in oil, coal, and tar deposits and are produced as by products of fuel burning (whether fossil fuel or biomass). PAHs are one of the most important groups of pollutants and have been identified to be teratogenic, carcinogenic, and mutagenic. Moreover, they have been shown to disrupt sex hormones, affect immunocompetence of organisms, and pose reproductive and developmental toxicity as well as skin defects in animals [2–4]. For the last few years, contamination of the environment has been a global problem and the effect of PAHs to the environment has raised a lot of concern. Therefore, the detection, identification, quantification and removal of PAHs are of considerable interest in environmental analysis. Several methods including immunoassay [5], gas chromatography [6] and high performance liquid chromatography (HPLC) with UV-vis absorbance or fluorescence detection [7,8] and capillary electrophoresis (CE) equipped with laser-induced fluorescence [9] have been used for the determination of PAHs. The drawbacks of these methods although the most accurate, are that they are expensive, time consuming, require large sample volumes as well as large amount of organic solvent with separation and extraction procedures, and must be undertaken by an analytical chemist in a dedicated analytical laboratory [10,11]. The aim of this work is to develop a simple and less time consuming electroanalytical method for the determination of one of the highly carcinogenic polyaromatic hydrocarbons namely, anthracene.

At the present time, electrochemistry is considered as one of the most efficient solution to environmental problems because a completely clean reagent, *i.e.*, the electron, is utilized [12]. The application of metal nanoparticles has attracted a lot of interest in the field of electrochemistry because of their extreme small size, high specific surface area, high surface to volume ratio and unique physicochemical characteristics [13] and their use in the fabrication of electrochemical devices is a promising prospect. They hold important application as catalysts. One of the greatest challenges faced in the use of electroanalytical methods in the determination of PAHs is electrode fouling which hinders stability of the methods. However, to avoid electrode fouling, the incorporation of overoxidized-polypyrrole which is known to prevent electrode fouling together with the bimetallic nanoparticles is necessary for the electro oxidation of PAHs. Polypyrrole (PPy) which is a conducting polymer has many attractive features as a molecular recognition system and can further be overoxidized at a high positive potential, expelling the doping ions and losing its conductivity as well as creating some nanopores on the polymer which enhances the immobilization of nanoparticles [14]. This article thus describes the use of overoxidized-polypyrrole/Ag-Au bimetallic nanoparticles composite (PPyox/Ag-AuNPs) in the development of an electrochemical sensor for the determination of one of the priority polyaromatic hydrocarbons, anthracene.

## 2. Experimental Section

### 2.1. Reagents and Materials

Analytical grade pyrrole (Py, 99%), hydrogen tetrachloroaurate (III) trihydrate (HAuCl_4_·3H_2_O), trisodium citrate (Na_3_C_6_H_5_O_7_, 99%), silver nitrate (AgNO_3_, 99%), anthracene (99%), lithium perchlorate (LiClO_4_, 99.99%), acetonitrile (HPLC grade) were obtained from Sigma-Aldrich. All chemicals were used as received except pyrrole which was double distilled under nitrogen before use. Ultra pure water (Millipore) was used for all preparations. Anthracene working solutions were prepared in acetonitrile.

### 2.2. Measurement and Instrumentation

Voltammetric measurements were performed on a BAS 50B electrochemical analyzer from Bioanalytical systems inc. (West Lafayette, IN) with conventional three-electrode system consisting of glassy carbon (GCE), Ag/AgCl (saturated NaCl) and platinum wire as working, reference and counter electrodes respectively. Lithium perchlorate was used as the supporting electrolyte. All experimental solutions were purged with high purity argon gas and blanketed with argon atmosphere during measurements. The experiments were carried out at controlled room temperature (25 °C). UV-Vis spectra measurements were recorded with the Nicolette Evolution 100 Spectrometer (Thermo Electron Corporation, UK). Transmission electron microscopy (TEM) images were acquired using a Tecnai G^2^ F_2_O X-Twin MAT. TEM characterizations were performed by placing a drop of the solution on a carbon coated copper grid and dried under electric bulb for 30 min. Fluorescence spectra measurements were obtained by the use of the Horiba Jobin Yvon NanoLog™.

### 2.3. Preparation of Monometallic Silver Nanoparticles, Gold Nanoparticles and Silver-Gold Alloy Nanoparticles

Silver nanoparticles (AgNPs) were synthesized by the reduction of AgNO_3_ with trisodium citrate according to a procedures described by Fang *et al.* [15] and Asta *et al*. [16]. Briefly, 50 mL of 1.0 mM AgNO_3_ solution was heated to boiling in an Erlenmeyer flask. To this solution, 5 mL of 1% C_6_H_5_O_7_Na_3_ was added dropwise. During the process, the solution was mixed vigorously by the use of a magnetic stirrer. The solution was heated until a pale yellow colour was observed. It was then removed from the heating surface and stirred until it cooled to room temperature. The mechanism of the reaction can be expressed as follows [16]:

(1) $${4\;\text{Ag}}^{+}{+\;C}_{6}H_{5}O_{7}{\text{Na}}_{3}{\;+\;2\;H}_{2}O\to {4\;\text{Ag}}^{0}{+C}_{6}H_{5}O_{7}H_{3}{+3\;\text{Na}}^{+}{+\;H}^{+}{+O}_{2}$$

Gold nanoparticles (AuNPs) were prepared through the reduction of 1.0 mM HAuCl_4_ using sodium citrate as the reducing agent. Twenty mL of 1.0 mM HAuCl_4_ solution was added to a 50 mL Erlenmeyer flask on a stirring hot place. A magnetic stirrer was added into the solution and the solution heated to boil. To the boiling solution, 2 mL of 1% solution of trisodium citrate dihydrate, Na_3_C_6_H_5_O_7_·2H_2_O was added. Gold sol gradually formed as the citrate reduced Au^3+^ to Au^0^. The solution was heated until a deep red colour was observed [17].

Silver-gold alloy nanoparticles (Ag-AuNPs) were synthesized via a previously reported procedure [18]. Briefly, 49 mL of water was added into a 100 mL round bottomed flask. 2% (w/v) sodium citrate (0.5 mL) was added into the water and the reaction mixture heated to 92 °C. A mixture of 10 mM HAuCl_4_ and 10 mM AgNO_3_ solution (0.5 mL) was added into the reaction mixture and the temperature regulated between 90 °C and 92 °C and refluxed for 1 hour. The volume of the mixture was adjusted so as to prepare Ag-Au with Ag/Au ratio of 1:3 by mixing 0.125 mL of 10 mM AgNO_3_ and 0.375 mL 10 mM HAuCl_4_ and the mixture maintained at 0.5 mL. There was a colour change observed in the solution (dark red) indicating the formation of nanoparticles according to the hallmarks reported in the literature [19].

### 2.4. Characterization of the Ag-Au Bimetallic Nanoparticles

Synthesized bimetallic Ag-Au nanoparticles (Ag-AuNPs) were characterized using UV-vis, electrochemical impedance spectroscopy (EIS), TEM, cyclic voltammetry and fluorescence spectroscopy.

### 2.5. Fabrication of Over-Oxidized Polypyrrole/Ag-AuNPs Composite Film-Modified Electrode (PPyox/Ag-AuNPs/GCE)

Prior to modification, the bare GCE was polished to a mirror finish with 1.0, 0.3, and 0.05 μm alumina slurries, respectively, and then rinsed thoroughly with distilled water followed by sonication in ethanol and water, respectively. Electrochemical polymerization of pyrrole was carried out in 0.1 M LiClO_4_ solution containing 0.1 M pyrrole by cycling the potential from −400 to 700 mV at a scan rate of 50 mV s^−1^ for 10 cycles. The electrode was rinsed with water and then transferred into 0.1 M NaOH solution for electrochemical overoxidation of the conductive PPy film at +1.0 V for 7 min. The resulting modified electrode was ready for use after rinsing with water and it is henceforth denoted as PPyox/GCE. In order to incorporate the intermetallic nanoparticles, 4 μL solution of already synthesized Ag-Au alloy nanoparticles were drop-coated on the PPyox/GCE and allowed to dry at room temperature. The modified electrode was taken out and rinsed with water, and it is henceforth denoted as PPyox/Ag-AuNPs/GCE. For control purposes, Ag-modified and Au-modified electrodes as well as Ag-Au alloy nanoparticles modified electrodes were as well prepared by drop-evaporation over the bare GCE, and each is henceforth denoted as AgNPs/GCE, AuNPs/GCE and Ag-Au NPs/GCE, respectively.

## 3. Results and Discussion

### 3.1. Characterization of Silver-Gold Alloy Nanoparticles

**Figure 1.**
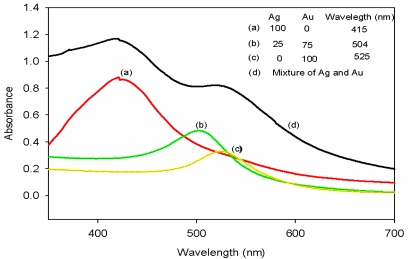
UV-vis absorption spectra of Ag (curve **a**), Au (curve **c**), Ag-Au alloy (curve **b**) nanoparticles and a mixture of pure Ag and Au nanoparticles (curve **d**).

The formation of silver-gold alloy nanoparticles by simultaneous reduction of gold and silver ions was confirmed by the use of UV-visible spectroscopy. Figure 1 shows the visible absorption spectra of gold, silver, silver-gold bimetallic alloy nanoparticles and a mixture of silver and gold nanoparticles. Absorption maximum for silver and gold nanoparticles were observed at 415 nm and 525 nm, respectively. The Ag-Au bimetallic alloy nanoparticles showed an absorption peak at 504 nm. The appearance of single absorption peaks at 504 nm which was between 525 nm and 400 nm shows that Ag-Au alloy nanoparticles were formed [18–20].

When pure gold and silver nanoparticles were mixed physically, two absorption peaks were observed (curve d). However, only one absorption peak was observed (curve b) for the solution of the bimetallic alloy nanoparticles. This is a confirmation that the synthesized nanoparticles were alloys and not mixture of elemental nano-particles as described by Pal *et al*. [21].

**Figure 2.**
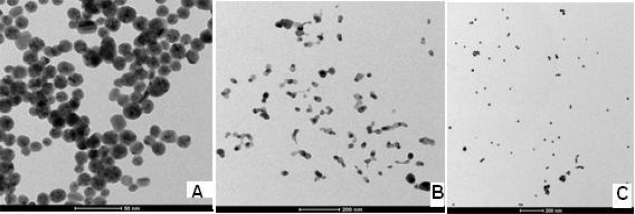
TEM image of **(A)** Ag-Au bimetallic alloy nanoparticles, **(B)** Ag nanoparticles and **(C)** Au nanoparticles.

Alloy formation can be attributed to similar lattice constants of 0.408 and 0.409 nm, respectively, for gold and silver. This small difference in lattice constants, being smaller than the amplitude of thermal vibrations of atoms, has already been hypothesized to favor alloy formation even at the nanometer scale [22]. The size and shape of the nanoparticles were determined by transmission electron microscopy. From Figure 2, particle sizes in the range of ca 20–30 nm, 30–40 nm and 20–30 nm were observed for Ag-Au, Ag and Au nanoparticles, respectively. The nanoparticles stored in an air-sealed bottle at a temperature of 25 ± 2 °C were found to be very stable, since no apparent change was observed by UV-visible absorption spectra even after about four months.

### 3.2. Fluorescence Properties of Silver-Gold Bimetallic Alloy Nanoparticles

**Figure 3.**
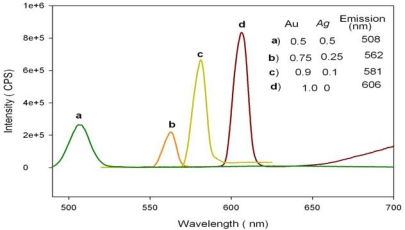
Emission spectra of Au and Ag-Au alloy (Au mole fraction: 0.5, 0.75, 0.9 and 1) nanoparticles with varying Au mole fraction.

The characteristic fluorescence property of silver-gold bimetallic alloy nanoparticles was observed by measuring fluorescence emission spectra. The fluorescence properties of silver and gold nanoparticles are attributed to electronic transitions between the highest valency d-band and conductance sp-band [18]. The fluorescence spectra of Ag-Au alloy nanoparticles are given in Figure 3. The emission spectrum (fluorescence band) for pure Au nanoparticles was observed at 606 nm when excitation was carried out at 525 nm. Ag-Au bimetallic alloy nanoparticles showed a fluorescence band at 562 nm when excitation was carried out at 504 nm. The concentration of the gold content and silver content in the alloy composition was varied and spectra were recorded for each case (see Figure 3).

It is worth noting that with silver concentration above 50% in the alloy, we were not able to observe any emission spectra after exciting the alloy nanoparticles at their respective, *λ*_max_ values. The fluorescence properties of silver and gold nanoparticles have been attributed to electronic transitions between the highest valence d-band and conductance sp-band [23]. The d-band electrons of the noble metal nanoparticles absorb the incident photon energy and get promoted to higher electronic states in the sp-band. The electron-hole pair recombines non-radiatively through electron-photon scattering process but then may combine radiatively giving rise to the observed fluorescence [21]. So, as the Au content in the alloy increases the energy gap between valence and conduction band decreases and as a result the emission peaks of the alloy nanoparticles showed an increase in wavelength [18]. Thus, the Ag-Au bimetallic alloy was confirmed to have been formed.

### 3.3. Electrochemical Characterization of Ag-Au Alloy Nanoparticles and PPyox/Ag-AuNPs/GCE

#### 3.3.1. Characterization of Ag-AuNPs/GCE in Neutral Medium

**Figure 4.**
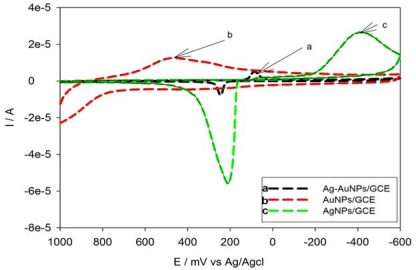
Cyclic voltammograms of the overlay of Ag-AuNPs/GCE (curve **a**), AuNPs/GCE (curve **b**) and AgNPs/GCE (curve **c**) in 0.1 M phosphate buffer pH 7; Scan rate 50 mV s^−1^.

The synthesized Ag-Au bimetallic alloy nanoparticles were also characterized by the use of cyclic voltammetric technique. Four μL solutions of the synthesized Ag-Au alloy nanoparticles was drop-coated on the surface of the polished GCE, allowed to dry at room temperature and rinsed with water in order to remove any loosely adsorbed nanoparticles. Cyclic voltammetry (CV) of Ag-AuNPs/GCE was then carried out in degassed 0.1 M phosphate buffer solutions, pH 7, at a scan rate of 50 mV s^−1^. This was compared with AgNPs/GCE and AuNPs/GCE and an overlay of the CVs done as shown in Figure 4.

Large irreversible oxidation and reduction peaks of Ag nanoparticles were observed around −414 mV and 209 mV (corresponding to the Ag/Ag^+^ redox couple), respectively (see Figure 4 (curve c)). The Au nanoparticles-modified electrodes also showed a broad anodic peak and less pronounced reduction peak corresponding to the oxidation and reduction of Au at a potential range of 0 to 700 mV (Figure 4 (curve b)). For the Ag-Au NPs/GCE, a reduction peak was observed around 86 mV while an anodic peak was observed at 245 mV (Figure 4 (curve a)). It is interesting to note that, for the Ag-Au alloy nanoparticles, only one reduction and one oxidation peak at a potential range of 0 to 400 mV was observed. This showed that the Ag-Au alloy nanoparticles were composed of atomically mixed Ag and Au atoms and not composed of Ag and Au metal domains. The concentration of the alloy nanoparticles deposited on the GCE was calculated to be 1.186 × 10^−11^ g.

#### 3.3.2. Electrochemical Impedance Spectroscopy (EIS) of the PPyox/Ag-AuNPs/GCE

The preparation process of electrodes was also monitored by electrochemical impedance spectroscopy (EIS), which is an effective method for probing the features of the surface of modified electrodes [24–26]. Electrochemical impedance spectra (EIS) measurements were performed in KCl (0.1 M) solutions containing [Fe(CN)_6_]^3−/4−^ and plotted in the form of complex plane diagrams (Nyquist plots) with a frequency range of 100 kHz to 0.1 Hz. The amplitude of the applied sine wave potential was 10 mV, whereas the ambient applied dc potentials were set at the formal potentials which were obtained from the CV experiments of the [Fe(CN)_6_]^3−/4−^ redox probe.

**Figure 5.**
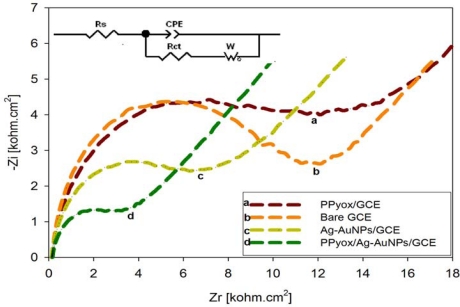
Nyquist plots of the EIS recorded in the presence of [Fe(CN)_6_]^3−/4−^ redox system in aq. KCl (0.1 M) for the PPyox/GCE (curve **a**), bare GCE (curve **b**), Ag-AuNPs/GCE (curve **c**) and PPyox/Ag-AuNPs/GCE (curve **d**).

From Figure 5, significant difference of the charge transfer resistances (*R*_ct_s) was observed upon the stepwise formation of the modified electrodes. The *R*_ct_ value for the bare GCE (curve b) was 9.125 × 10^3^ Ω. After the PPyox film was deposited on the GCE, the *R*_ct_ value was found to be 9.684 × 10^3^ Ω (curve a). As compared to curve b, the *R*_ct_ of curve a increased. This can be attributed to the negatively charged and the low conductive PPyox film [27]. The negative charge of the layer should be unfavorable for the approaching of ferricyanide anions. After the bare GCE was modified with Ag-AuNPs, the *R*_ct_ value (4.674 × 10^3^ Ω) decreased obviously (curve c) implying that the Ag-AuNPs played an important role in accelerating the transfer of electrons. For the PPyox/Ag-AuNPs/GCE, the *R*_ct_ value (2.800 × 10^3^ Ω) decreased further (curve d) an indicator that the composite was more facile to the electron transfer.

#### 3.3.3. Cyclic Voltammetric Behaviour of the PPyox/Ag-AuNPs/GCE in the Presence of K_3_Fe(CN)_6_

**Figure 6.**
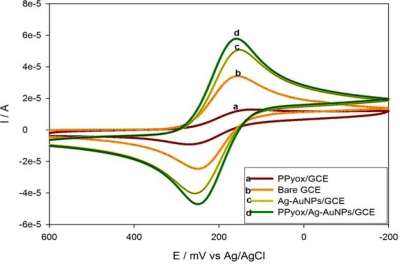
CVs of PPyox/GCE (curve **a**), bare GCE (curve **b**), Ag-AuNPs/GCE (curve **c**) and PPyox/Ag-AuNPs/GCE (curve **d**) in 5 mM K_3_Fe(CN)_6_ solution containing 0.1 M KCl. Scan rate: 50 mV s^−1^.

Cyclic voltammetry (CV) of the ferricyanide system is a convenient and valuable tool to monitor the electrochemical characteristics of the surface of modified electrodes. CVs at the bare and the different modified electrodes in 5 mM K_3_Fe(CN)_6_ containing aq. KCl (0.1 M) were shown in Figure 6.

Overoxidized polypyrrole (PPyox) is prepared by oxidizing polypyrrole (PPy) to a higher oxidation state after which it becomes susceptible to nucleophilic attack [27]. This process usually results in the addition of carbonyl functionality to the pyrrolic rings with a consequent loss of conjugation and hence the loss of inherent electronic conductivity [27,28]. From Figure 6, curve a, PPyox modified GCE showed lower peak currents (65% decrease) and larger peak to peak separations (Δ*E*_p_ = 143) indicating that there was slower electron transfer compared to that recorded with the bare GCE (Figure 6 (curve b), Δ*E*_p_ = 91 mV). This can be attributed to the negatively charged and the low conductive PPyox film [27–30]. The negative charge of the layer should be unfavorable for the approaching of ferricyanide anions. After the Ag-AuNPs were assembled on the GCE (curve c), the peak current increased (54% increase) dramatically in comparison to that in curve b. The reason was that Ag-AuNPs with large specific surface area and good conductivity could act as conduction centers which facilitate the transfer of electrons, so it could accumulate much more K_3_Fe(CN)_6_ on the modified electrode. However, the purpose of the overoxidizing the PPy was to exploit the fact that this process results in a polymer with pores on its surface, which creates a better condition for the attachment of nano-particles [31]. According to the cyclic voltammograms of the K_3_Fe(CN)_6_ redox probe shown in Figure 6, after deposition of the Ag-Au alloy nano-particles in the presence of electrodeposited PPyox-film on the GCE’s surface (curve d), the peak current was enhanced by over 100% and the peak-to-peak separation (Δ*E*_p_ = 90) was reduced by 53 mV when compared to the CV recorded with PPyox film-modified electrode without the Ag-AuNPs (see data in Table 1). Relative to the bare GCE, the increased surface roughness and the newly induced surface functional groups and micro/nano catchment sites of the PPyox/GCE might have enhanced the loading of the nano-particles and, thus, created significant change in the surface area and the kinetics involved. This agrees well with the results presented in Figure 5.

### 3.4. Electrocatalytic Oxidation of Anthracene on PPyox/Ag-AuNPs/GCE

**Figure 7.**
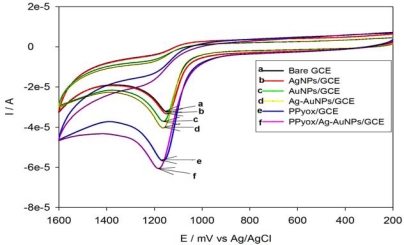
CV of 3.56 × 10^−4^ M anthracene at bare/GCE (curve **a**), AgNPs/GCE (curve **b**), AuNPs/GCE (curve **c**), Ag-AuNPs/GCE (curve **d**), PPyox/GCE (curve **e**) and PPyox/Ag-AuNPs/GCE (curve **f**) in acetonitrile and 0.1 M LiClO_4_ at a scan rate of 100 mV s^−1^.

Experiments on the electrocatalytic oxidation of anthracene were investigated with different modified electrodes in acetonitrile with 0.1 M LiClO_4_ as the supporting electrolyte. The electrochemical processes observed at all electrodes were only for the oxidation of anthracene. The following discussion is based on their cyclic voltammetric responses (see Figure 7) to anthracene (3.56 × 10^−4^ M) dissolved in the above solution.

When using PPyox/Ag-Au alloy nanoparticles modified electrode (curve f), the anodic peak observed was 88.63% higher than that for the bare electrode. However, the AgNPs/GCE, AuNPs/GCE, Ag-AuNPs/GCE and PPyox/GCE exhibited peak enhancement of 4.69%, 15.27%, 28.88% and 68.12% respectively compared to the bare GCE (see Table 2 for summary of CV characteristics).

The better electrocatalytic performance of the AgNPs/GCE, AuNPs/GCE and Ag-AuNPs/GCE as measured with their higher amperometric sensitivities than the bare GCE might be attributed to the presence of nanoparticles on GCE. According to Figure 7, the electrode which was modified with the bimetallic alloy nanoparticles exhibited higher catalytic properties compared to the monometallic nanoparticles modified electrodes. A similar observation was reported in the literature [32]. However, the contribution of modifying the GCE with PPyox as represented by “curve e” was most dominant as the PPyox/GCE alone exhibited about 70% increase in the anodic peak compared to the bare electrode. On deposition of the bimetallic Ag-Au nanoparticles over the PPyox/GCE, the anodic peak was observed to slightly increase to about 84% as shown by the response of the PPyox/Ag-AuNPs/GCE by “curve f”. The catalytic effect of combining PPyox with Ag-AuNPs as evidenced by the remarkable enhancement in the peak height for anthracene oxidation might be attributable to the adsorptive ability of PPyox film and its high nanoparticle-loading nano-porous structure allowing the immobilization of the Ag-AuNPs which have high catalytic properties.

**Figure 8.**
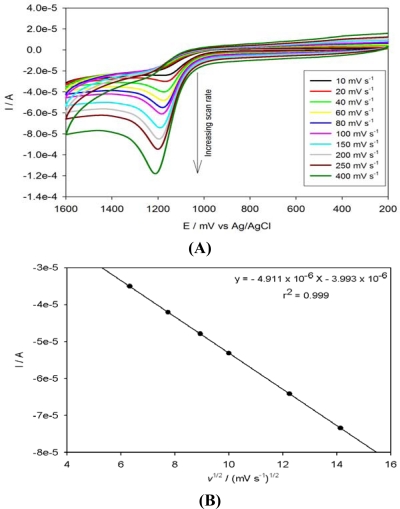
CVs of PPyox/Ag-AuNPs/GCE upon addition of 3.56 × 10^−4^ M anthracene at different scan rates **(A)** and a plot of root scan rate *versus* peak current **(B)**.

The effect of potential scan rate was investigated. Increasing the scan rates caused an increase in the peak current. Figure 8(A) shows the CV of PPyox/Ag-AuNPs/GCE upon addition of 3.56 × 10^−4^ M anthracene at different scan rates. The peak current for anthracene increased linearly with the square root of the sweep rate (*ν*^½^) over the range 20–250 mV s^−1^ with a linear regression of *I*_pa_ (A) = −4.911 × 10^−6^ − 3.993 × 10^−6^ *ν*^½^(mV s^−1^)^½^ (r^2^ = 0.999) (Figure 8(B)). Thus, the electrochemical process was limited by the rate of diffusion of anthracene from the solution to the electrode surface. Moreover, on increasing the scan rate, the magnitude of the peak current increased. However, the peak potentials of all the peak potentials shifted to more positive potentials. This also confirmed that the peak currents were diffusion-controlled [33].

### 3.5. Analytical Application for Anthracene Detection

**Figure 9.**
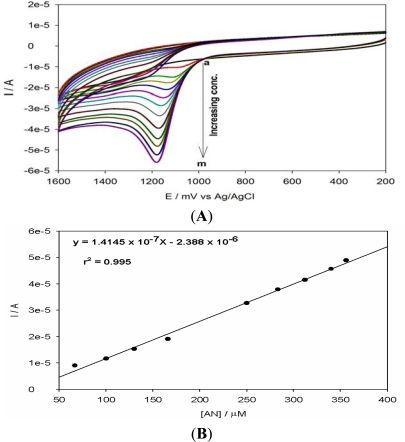
**(A)** CV of PPyox/Ag-AuNPs/GCE in acetonitrile +0.1 M LiClO_4_ with different anthracene concentration: (a) 0, (b) 33, (c) 66, (d) 100, (e) 133, (f) 166, (g) 200, (h) 233, (i) 250, (j) 283, (k) 312, (l) 340, (m) 356 μM at a scan rate of 100 mV s^−1^ and (**B**). A calibration plot showing the relationship between oxidation current and concentration of anthracene.

The cyclic voltammograms of PPyox/Ag-Au-modified electrode in acetonitrile solution of LiClO_4_ (0.1 M) containing different concentrations of anthracene are shown in Figure 9(A). A calibration curve (Figure 9(B)) based on the anodic peak heights from Figure 9(A), plotted against concentration, was found to be satisfactorily linear (r^2^ = 0.995) for analytical application of this sensor. The anodic peak potential of anthracene at the PPyox/Ag-Au film modified electrode was at about 1,181 mV [34].

**Figure 10.**
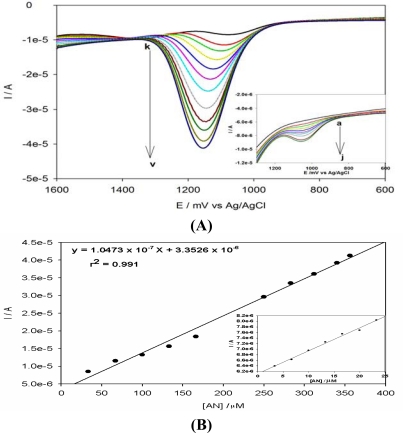
**(A).** Anodic difference SWV of PPyox/Ag-AuNPs/GCE in acetonitrile +0.1 M LiClO_4_ with different anthracene concentration: (a) 0, (b) 3, (c) 6, (d) 10, (e) 13, (f) 16, (g) 20, (h) 23, (i) 26, (j) 28, (k) 33, (l) 66, (m) 100, (n) 133, (o) 166, (p) 200, (q) 233, (r) 250, (s) 283, (t) 312, (u) 340, (v) 356 μM. Inset shows the curves at low concentrations. **(B)**. A calibration plot showing the relationship between current and concentration of anthracene. Inset shows a calibration plot at low concentration.

This was confirmed by the use of SWV in Figure 10(A) which showed similar results. Lower concentrations of anthracene were also determined using SWV (Figure 10(A) inset) and a calibration plot drawn as shown in Figure 10(B). Based on the SWV measurements, a linear relationship between the anodic current and anthracene concentration was attained over the range of 3.0 × 10^−6^ to 3.56 × 10^−4^ M with a correlation coefficient of 0.991. The limit of detection for anthracene from standard solutions, calculated as the concentration corresponding to the signal three times the standard deviation of blank measurements was 1.69 × 10^−7^ M.

**Figure 11.**
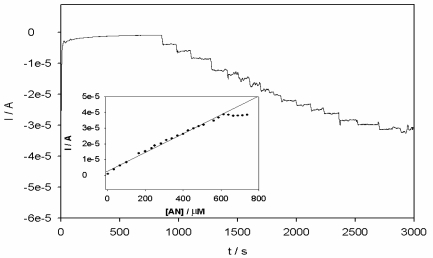
Current-time plot for the steady state response of the PPyox/Ag-AuNPs/GCE upon the successive addition of 3.0 μM anthracene at 1,181 mV. The measurements were carried out in a continuously stirred acetonitrile solution of LiClO_4_ (0.1 M). The inset shows a calibration plot of current against concentration.

While the above calibration was based on kinetic principle, it would also be interesting to see how the electrode would perform under steady state condition. Figure 11 shows the steady state amperometry response of sensor for successive additions of anthracene (3.0 μM each time) into the acetonitrile and 0.1 M LiClO_4_ solution sequentially.

From the steady state amperogram (Figure 11), successive additions of anthracene resulted in favorable responses on the PPyox/Ag-AuNPs/GCE achieving steadystate within 4 s. The linear dependence of current response on the anthracene concentration as shown in the calibration curve (Figure 11 inset) was linear in the range 3.0 × 10^−6^ to 5.9 × 10^−4^ M (r^2^ = 0.992).

### 3.6. Reproducibility, Stability and Interference Studies

The stability and reproducibility are key elements of electrode performance. The reproducibility of the PPyox/Ag-Au nanoparticles modified electrode was investigated in the presence of 3.56 × 10^−4^ M anthracene in acetonitrile and 0.1 M LiClO_4_. Square wave voltammetric experiments were repeatedly performed for 6 times with the same PPyox/Ag-Au nanoparticles modified electrode in the solution of 3.56 × 10^−4^ M anthracene. The relative standard deviation was 1.2% (n = 6) confirming that the results were reproducible. The stability of PPyox/Ag-AuNPs/GCE sensor was investigated by measuring the electrode response with 3.56 × 10^−4^ M anthracene after every 4 days by SWV. It was found that there was almost 19.26% decrease of the peak current response on the modified electrode for 3.56 × 10^−4^ M after 2 weeks thus the modified electrode retained 80.74% of its initial response after it was kept in refrigerator at 4 °C for 14 days. This can be attributed to the excellent stability of the film. Inorganic ions which are likely to be present in water samples especially water were investigated as possible sources of interferences. No interference could be observed for the following ions: Na^+^, Cu^2+^, Fe^2+^, Mn^2+^, Cl^−^, SO_4_^2−^, and NO_3_^−^ where the anthracene concentration was 3.56 × 10^−4^ M.

## 4. Conclusions

Ag-Au bimetallic nanoparticles/overoxidized-polypyrrole composite exhibited remarkable electrocatalytic activity towards anthracene oxidation, improving the rate of electrochemical oxidation of anthracene and amperometric response sensitivity of the system to the same. As one of the challenges faced when constructing electrochemical sensors for the detection of polyaromatic hydrocarbons is electrode fouling, the fact the sensor reported here showed no electrode fouling is a significant outcome of our research. The method was rapid, sensitive, and inexpensive and did not necessitate great amounts of organic compounds. The designed sensor can be applied in the detection of anthracene in water due its low detection limits and sensitivity. A further study is underway regarding ways of improving its detection limit and the possible application of the sensor in the detection of anthracene in waste water.

## Figures and Tables

**Table 1.** CV data obtained from Figure 6.

**Electrode**	***E*_pa_ (mV)**	***E*_pc_ (mV)**	***I*_pa_ (A)**	***I*_pc_(A)**	**Δ*E*_p_**	***E*^o^'**
PPyox/GCE	264	125	−1.189 × 10^−5^	1.170 × 10^−5^	143	194.5
Bare GCE	248	157	−3.328 × 10^−5^	3.357 × 10^−5^	91	202.5
Ag-AuNPs/GCE	255	152	−5.103 × 10^−5^	5.186 × 10^−5^	103	203.5
PPyox/Ag-AuNPs/GCE	249	159	−5.919 × 10^−5^	5.939 × 10^−5^	90	204.0

**Table 2.** Anodic current response data for the development of the sensor (see Figure 7).

**Electrode**	***E*_pa_ (mV)**	***I*_pa_ (A)**
Bare GCE	1,148	2.778 × 10^−5^
AgNps/GCE	1,150	2.900 × 10^−5^
AuNPs/GCE	1,160	3.193 × 10^−5^
Ag-AuNPs/GCE	1,162	3.537 × 10^−5^
PPyox/GCE	1,162	4.657 × 10^−5^
PPyox/Ag-AuNPs/GCE	1,181	5.225 × 10^−5^
